# CT differentiation of gastric ectopic pancreas from gastric stromal tumor

**DOI:** 10.1186/s12876-021-01617-8

**Published:** 2021-02-04

**Authors:** Chang Liu, Fang Yang, Wenming Zhang, Weiqun Ao, Yongyu An, Cui Zhang, Bailing Dai, Cailing Pu, Jian Wang

**Affiliations:** 1 Department of Radiology, The First Affiliated Hospital, College of Medicine, Zhejiang University, #79 Qingchun Road, Hangzhou, 310003 Zhejiang Province P. R. China; 2 Department of Pathology, Sir Run Run Shaw Hospital, Zhejiang University School of Medicine, 3 East Qingchun Road, Hangzhou, 310016 China; 3 Department of Radiology, Sir Run Run Shaw Hospital, Zhejiang University School of Medicine, 3 East Qingchun Road, Hangzhou, 310016 China; 4 Department of Radiology, TongDe Hospital of ZheJiang Province, No. 234, Gucui Road, Hangzhou, 310012 Zhejiang Province China

**Keywords:** Gastric ectopic pancreas, Gastric stromal tumor, Subepithelial tumors, Gastric neoplasm, Computed tomography

## Abstract

**Background:**

Gastric ectopic pancreas (GEPs) is a rare developmental anomaly which is difficult to differentiate it from submucosal tumor such as gastric stromal tumor (GST) by imaging methods. Since the treatments of the GEPs and GST are totally different, a correct diagnosis is essential. Therefore, we retrospectively investigated the CT features of them to help us deepen the understanding of GEPs and GST.

**Methods:**

This study enrolled 17 GEPs and 119 GST, which were proven pathologically. We assessed clinical and CT features to identify significant differential features of GEPs from GST using univariate and multivariate analyses.

**Results:**

In univariate analysis, among all clinicoradiologic features, features of age, symptom, tumor marker, location, contour, peritumoral infiltration or fat-line of peritumor, necrosis, calcification, CT attenuation value of unenhancement phase/arterial phase/portal venous phase (CTu/CTa/CTp), the CT attenuation value of arterial phase/portal venous phase minus that of unenhanced phase (DEAP/DEPP), long diameter (LD), short diameter (SD) were considered statistically significant for the differentiation of them. And the multivariate analysis revealed that location, peritumoral infiltration or fat-line of peritumor, necrosis and DEPP were independent factors affecting the identification of them. In addition, ROC analysis showed that the test efficiency of CTp was perfect (AUC = 0.900).

**Conclusion:**

Location, the presence of peritumoral infiltration or fat-line of peritumor, necrosis and DEPP are useful CT differentiators of GEPs from GST. In addition, the test efficiency of CTp in differentiating them was perfect (AUC = 0.900).

## Background

Ectopic pancreas, which was first described by Klob in 1859, is a rare developmental anomaly that is defined as pancreatic tissue lacking anatomic or vascular continuity with the main body of the gland [1–3]. It can occur anywhere along distal end of the esophagus to the colon, and of the gastrointestinal lesions, stomach (30%) is the most common area, followed by duodenum (25%) and jejunum (15%) [3]. Ectopic pancreas is usually discovered incidentally during surgery or autopsy and is generally asymptomatic. However, some patients with ectopic pancreas may have clinical symptoms such as abdominal pain, gastrointestinal bleeding, and obstruction when complicated with pancreatitis, pseudocyst, insulinoma, and pancreatic cancer [2, 4–7].

Although there were many diagnostic imaging methods such as computed tomography (CT), ultrasonography and endoscopic ultrasonography, the diagnosis of ectopic pancreas was difficult due to its non-specific imaging characteristics. Ectopic pancreas may commonly present as a submucosal mass in gastrointestinal tract [8] and were easily misdiagnosed with other submucosal tumor such as gastric stromal tumor (GST) and leiomyoma. As we know, GST is the most common subepithelial lesion and accounts for 90% of gastric submucosal tumors [8, 9]. Additionally, the standard treatment for a GST without metastasis is surgical resection and a GST with metastasis is usually treated by tyrosine inhibitors such as imatinib. However, an ectopic pancreas would not need clinical treatment unless it is symptomatic, in addition, whether to remove suspected ectopic pancreas that is found incidentally is still a controversial issue [10]. So it is of great clinical significance to identify ectopic pancreas and gastrointestinal stromal tumors on image.

As we know, CT is the most commonly used noninvasive modality for preoperative evaluation of gastric tumors due to its well-standardized protocal and easy accessibility. However, CT features of gastric ectopic pancreas (GEPs) as well as differential imaging features of GEPs from GST have not yet been investigated completely. Therefore, our study retrospectively investigates the differential CT features of GEPs from GST.

## Methods

### Patients

Our retrospective study was approved by the ethics commitee of Tongde hospital of Zhejiang Province and Sir Run Run Shaw Hospital, Zhejiang University School of Medicine3 and did not require informed consent. All procedures performed in studies involving human participants were in compliance with the 1964 Helsinki Declaration and its later amendments.

**Fig. 1:**
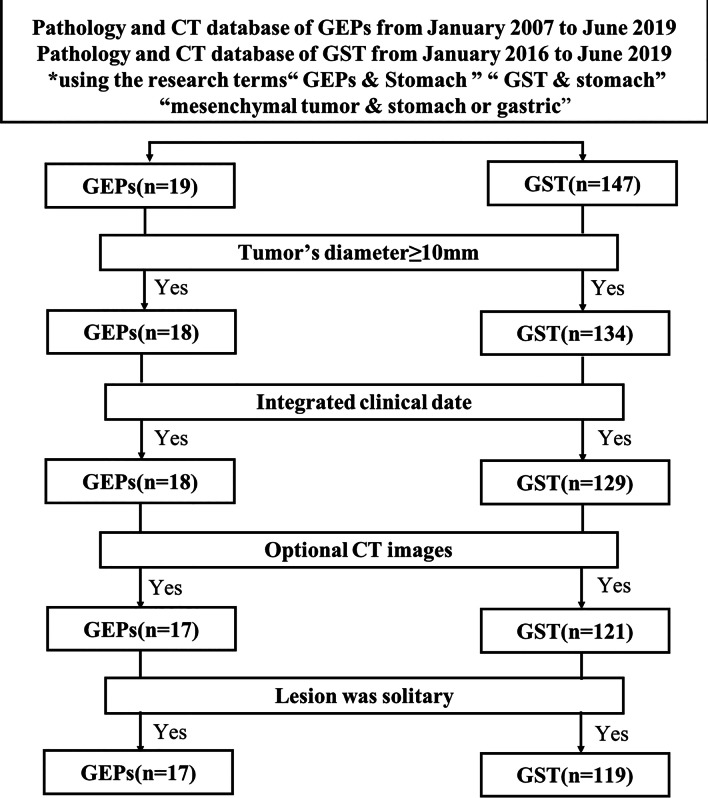
Flowchart of study base on recommended standards for differentiating diagnosis accuracy

We queried pathology database of our institute to derive all histologically proved cases of GEPs from January 2007 to June 2019 and GST from January 2016 to June 2019. All patients were histologically proven by surgical resection. Finally total 19 cases of GEPs and 147 cases of GST were identified in this query. We included patients who satisfied the following inclusion criteria: (1) patients who had preoperative CT images with optimal gastric distension, (2) patients who had integrated clinical date, (3) the lesion was solitary, (4) the maximum diameter of the tumor was no less than 10 mm. Finally, 17 patients with histopathologically proven GEPs and 119 patients with GST comprised our study population (Fig. 1).

### Clinical features

The clinical features of all patients were analyzed by one author (C.L) using the medical records of hospital. For each patient, the gender, age, the presence of clinical manifestations and the level of tumor marker (ferrithin) were analysed.

### CT image acquisition

All Enhanced CT images were obtained from multidetector CT scanners (SOMATOM, Sensation 16, Siemens, Forchheim, Germany and SOMATOM, Defnition AS+, Siemens, Forchheim, Germany). All patients drank 600–800 ml of water before CT examination. The CT scan parameters were set as follows: for SOMATOM Sensation 16, beam collimation = 1.2 mm × 16, pitch = 1, kVp/effective mA = 120/300, rotation time = 0.5 s and reconstruction section thickness = 5 mm; for SOMATOM, Defnition AS+, beam collimation = 1.2 mm × 32, pitch = 1, kVp/effective mA = 120/160, rotation time = 0.5 s, and reconstruction section thickness = 5 mm. Patients were injected with non-ionic contrast material (Ultravist; 300 mg I/ml, Bayer Schering Pharma AG, Berlin, Germany or Iopamidol; 300 mg I/ml, Bracco Sine Pharma AG, Shanghai, China) with antecubital venous access at a rate of 3.0 ml/sec and a total of 90–120 ml (1.5 mL per kilogram of body weight) was injected by using the CT-compatible power injector during arterial and parenchymal phase. The scanning delay for arterial imaging was determined by using automated scan-triggering software Arterial scanning automatically began 7.0 s after the trigger attenuation threshold (100 HU) was reached at the level of the superior abdominal aorta and parenchymal scanning began at a delay of 45 s after arterial scanning.

### Imaging analysis

All CT images were respectively interpreted by two radiologists (C.L. and J.W.) who had 4-yr (C.L.) and 12-yr (J.W.). Both radiologists were aware that the study population had either GEPs or GST, but were blinded to their histological subtypes. The following CT features of the primary gastric lesion were assessed: (1) the long diameter (LD) and short diameter (SD), (2) the ratio of Long diameter to short diameter (LD/SD), (3) location of the lesion (cardia; fundus; body; antrum), (4) contour of the lesion (round; oval; irregular), (5) Growth pattern (endophytic; exophytic; mixed), (6) the presence of peritumoral infiltration or fat-line of peritumor, wherein, the peritumoral infiltration was defined as a dense band-like perigastric fat infiltration; the fat-line of peritumor was defined as a fat space between the tumor and serosal layer, (7) the presence of necrosis, wherein, the necrosis was defined as the presence of non-enhancement low-density area within the tumor, (8) the presence of calcification, (9) the presence of surface ulceration, (10) the presence of lymph node, wherein, the lymph node was defined as the shortest axis length of the largest lymph node was more than 10 mm, (11) the CT attenuation value of unenhancement phase (CTu), (12) arterial enhancement (CTa) of the tumor, which measured the CT value at a represent region of interest (ROI), (13) parenchymal enhancement (CTp), (14) the CT attenuation value of arterial phase minus unenhancement phase (DEAP), (15) the CT attenuation value of portal venous phase minus unenhancement phase (DEPP).

### Statistical analysis

All statistical analyses were performed using commercial software, SPSS 22.0 for Windows (SPSS Inc, Chicago, IL, USA). The prevalence of clinical and CT features were compared using Student’s t-test and χ^2^ test, and binary logistic regression analyses were performed to achieve the most significant differential CT features. Receiver operating characteristic curve (ROC) analysis was performed to ascertain the optimal cut-off value of significant quantitative CT features, such as LD, SD, CTu, CTa, CTp, DEAP, DEPP and to obtain the value of sensitivity and specificity of qualitative CT features, such as location, contour, the presence of serosal invasion or fat-line of peritumor, necrosis and calcification. A *P* value < 0.05 was considered statistically significance.

## Result

### Clinical features

The clinical characteristics of GEPs and GST patients were summarized in Table 1. There was no significant difference in gender distribution between the two groups. However, there were significant differences in mean age, symptom and the level of serum tumor marker (*P* < 0.05).

**Table 1:** Clinical characteristics of 136 patients with GEPs and GST

Clinical characteristics	GEPs (n = 17)	GST (n = 119)	*P* value
Gender			0.948
Male	7 (41.2)	50 (26.3)	
Female	10 (58.8)	69 (73.7)	
Mean age (years, age range)	38.53 ± 2.468 (24–58)	59.46 ± 10.78 (29–88)	**< 0.001**
Symptom			0.001
Yes	3 (21.4)	70 (58.8)	
No	14 (78.6)	49 (41.2)	
Tumor marker^a^			0.013
Abnormal	1 (5.9)	43 (36.1)	
Normal	16 (94.1)	76 (63.9)	

*P* values written in bold indicate a significant difference between the tumors

*GEPs* gastric ectopic pancreas, *GST* gastrointestinal stromal tumor

^a^Abnormal ferritin level (excluding abnormal CA125 in the two GST)

### Qualitative and quantitative image analysis

Results of the qualitative and quantitative images analysis were presented in Table 2. The location of tumor was significantly different between the two groups (*P* < 0.05)—Or rather, there was a significant difference in the distribution of tumors in gastric fundus between two groups (*P* < 0.05). The majority of GEPs were located in body (64.7% [11/17]) and none (0% [0/17]) of GEPs were located in gastric fundus, whereas the majority of GST were located in body (47.1% [56/119]) and fundus (34.4% [41/119]). In addition, the contour of GEPs tended to be oval (58.8% [10/17]), while the contour of GST were round (30.3% [36/119]) or oval (30.3% [36/119]). Furthermore, GST were more likely to have the presence of peritumoral infiltration or fat-line of peritumor (*P* < 0.05). Moreover, there were significant differences in the necrosis, calcification, surface ulceration, lymph node. Although there wasn’t any significant difference about the tumor growth pattern, the GST were tended to grow exophytically.

**Table 2:** CT findings of 136 patients with GEPs and GST

CT features	GEPs (n = 17)	GST (n = 119)	*P* value	
			Univariate	Multivariate
Qualitative analysis				
Location			**0.006**	**0.021**
Cardia	1 (5.9%)	9(7.6%)	1.000	
Fundus	0 (0%)	41 (34.4%)	**0.004**	
Body	11 (64.7%)	56(47.1%)	0.173	
Antrum	5(29.4%)	13 (10.9%)	0.085	
Contour			**0.034**^a^	0.747
Round	5 (29.4%)	36(30.3%)	0.944	
Oval	10 (58.8%)	36(30.3%)	**0.020**	
Irregular	2 (11.8%)	47 (39.5%)	**0.026**	
Growth pattern			0.232^a^	
Endophytic	4 (23.5%)	57 (47.9%)	0.059	
Exophytic	11 (64.7%)	45 (37.8%)	**0.035**	
Mixed	2 (11.8%)	17 (14.3%)	1.000	
Peritumoral infiltration or fat-line of peritumor			< 0.001^a^	**0.001**
Yes	6 (35.3%)	3 (2.5%)		
No	11 (64.7%)	116 (97.5%)		
Necrosis	1 (5.9%)	41 (34.5%)	** < 0.001**^a^	**0.048**
Calcification	0 (0%)	23 (19.3%)	**0.048**^a^	0.211
Surface ulceration	0 (0%)	11 (9.2%)	0.193^a^	
Lymph node	0 (0%)	1 (0.8%)	1.000^a^	
Quantitative analysis				
CT attenuation value				
CTu (HU)	40.55 ± 8.67	34.24 ± 7.19	** < 0.001**^b^	0.600
CTa(HU)	71.35 ± 19.49	54.57 ± 13.77	**0.01**^b^	0.901
CTp(HU)	95.09 ± 13.15	68.92 ± 17.87	**0.003**^b^	0.752
Degree of enhancement				
DEAP	37.44 ± 15.70	20.37 ± 11.81	** < 0.001**^b^	**0.047**
DEPP	54.49 ± 17.69	34.26 ± 15.57	** < 0.001**^b^	0.762
LD (mm)	21.94 ± 11.81	35.26 ± 23.51	**0.001**^b^	0.097
SD (mm)	16.06 ± 6.07	28.55 ± 16.56	** < 0.001**^b^	0.069
LD/SD	1.329 ± 0.29	1.229 ± 0.21	0.07	

*P* values written in bold indicate a significant difference between the tumors. CTu/CTa/CTp = the CT attenuation value of unenhancement phase/arterial phase/portal venous phase; DEAP = the CT attenuation value of arterial phase—unenhanced phase; DEPP = the CT attenuation value of portal venous phase—unenhanced phase; LD = long diameter; SD = short diameter. Multivariate analysis was performed using logistic regression

^a^χ^2^ test

^b^Independent *t* test

**Fig. 2:**
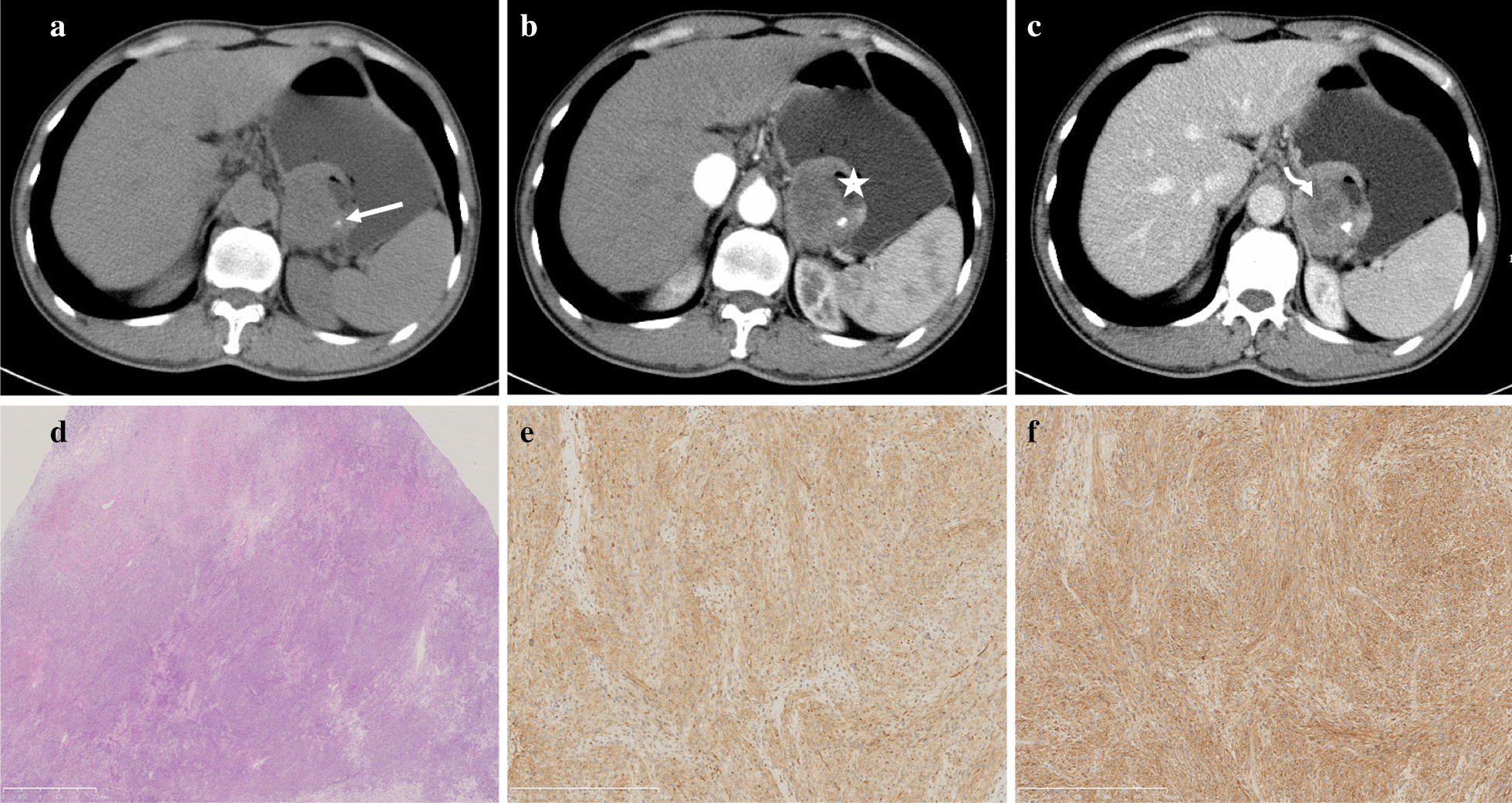
Patient 1, 50–60 years old, sex 1. Stromal tumor in gastric fundus (**a**–**c**). Axial CT scans (non-enhanced, arterial and portal phase) show an irregular mass with mixed growth pattern. Ulceration (*), calcification (arrow), necrosis (bend-arrow) are presented in the lesion and the mass shows mind to moderate heterogeneous enhancement (**d**–**f**). Histological and immunohistochemical images show that stromal tumor is positive for DOG1 (**e**) and CD117 (**f**)

**Fig. 3:**
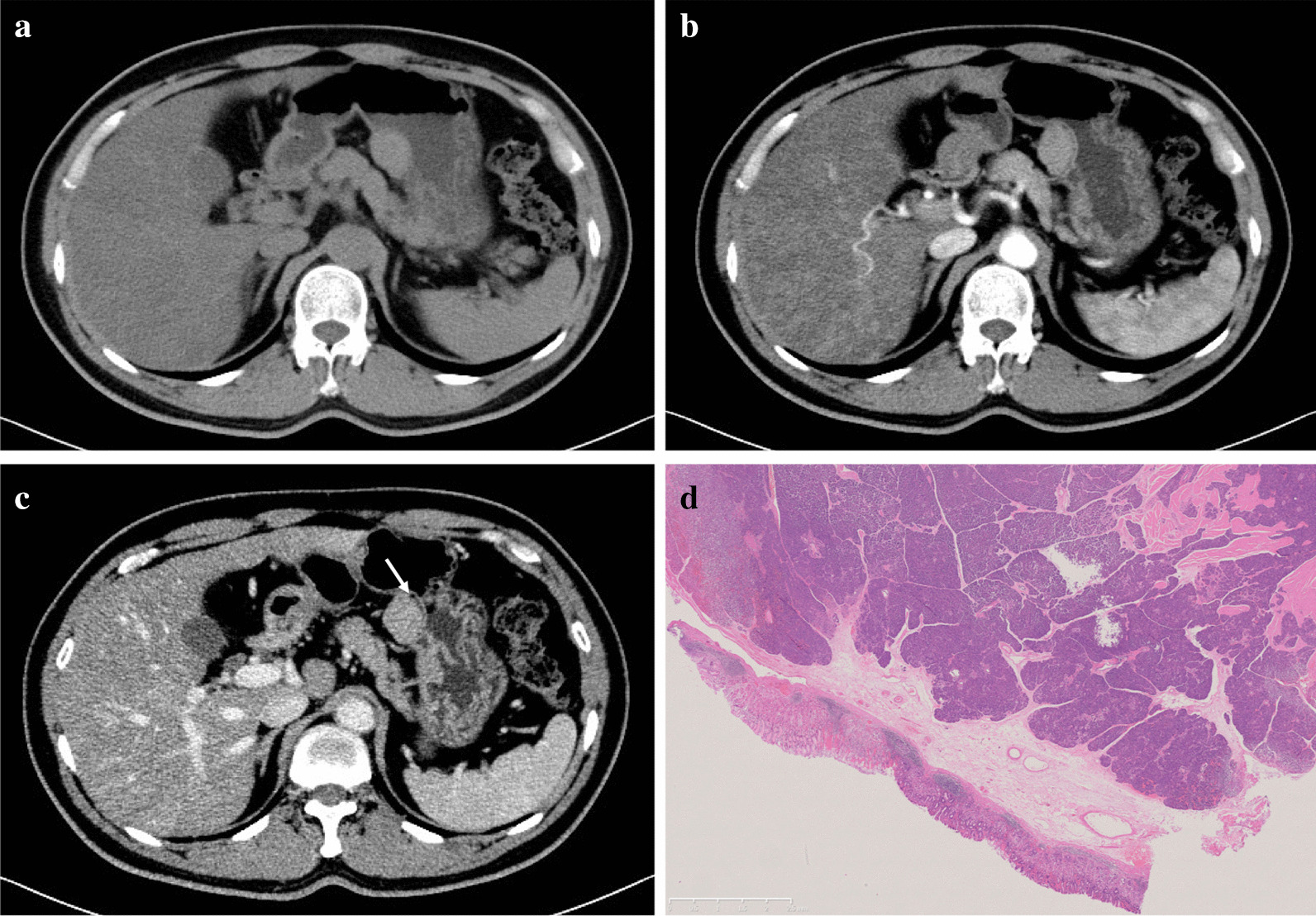
Patient 2, 40–50 years old, sex1. Ectopic pancreas in gastric body (**a**–**c**). Axial CT scans (non-enhanced, arterial and portal phase) show an oval Exophytic mass in the gastric body and also the fat-line of peritumor (arrow). The lesion shows homogeneous enhancement and equal attenuation to the pancreas (**d**). The lesion was confirmed as ectopic pancreas pathologically

When it comes to quantitative CT images analysis, the CT attenuation values such as CTu, CTa, CTp, DEAP, DEPP of GEPs were significantly higher (40.55HU ± 8.673for CTu, 71.35HU ± 19.49 for CTa, 95.09HU ± 13.15 for CTp, 37.44 HU ± 15.70 for DEAP and 54.49HU ± 17.69 for DEPP) than that of GST (34.24HU ± 7.19for CTu, 54.57HU ± 13.77 for CTa, 68.92HU ± 17.87for CTp, 20.37HU ± 11.81 for DEAP and 34.26HU ± 15.57for DEPP) (*P* < 0.05). Moreover, the LD and SD were also the significant CT features in differentiating GEPs from GST (*P* < 0.05). In addition, multivariate analysis revealed that location, the presence of peritumoral infiltration or fat-line of peritumor (Figs. 2, 3), necrosis and DEPP were independent factors affecting the identification of GEPs and GST.

### Sensitivity and specificity values for CT diagnosis

**Fig. 4:**
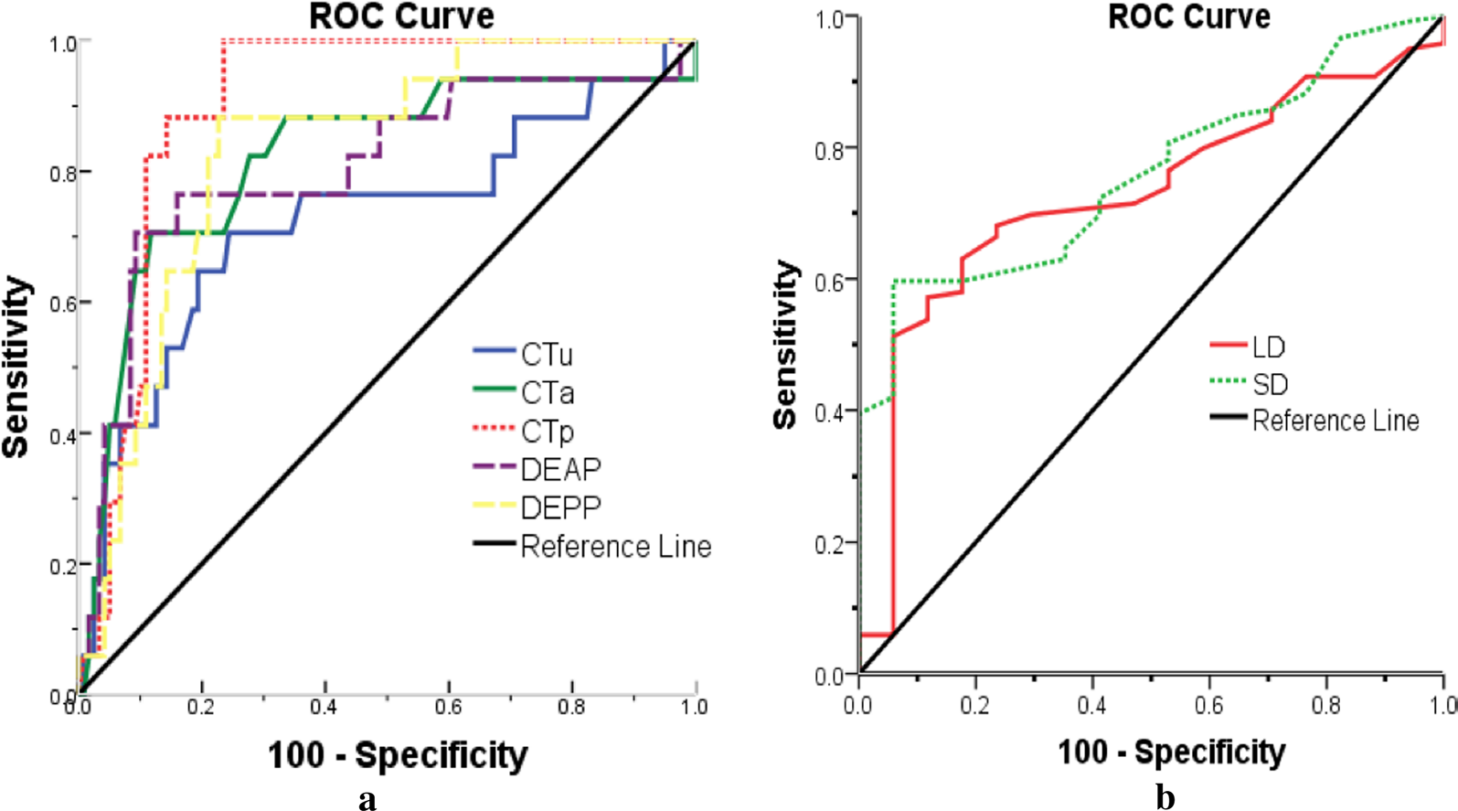
Receiver operating characteristic (ROC) curves of quantitative values to differentiate GEPs from GST. The largest area under the curve (AUC) was the CTp (0.900), followed by the DEPP (0.832), the CTa (0.821), the DEAP (0.806), the SD (0.757), the CTu (0.727) and the LD (0.726)

**Fig. 5:**
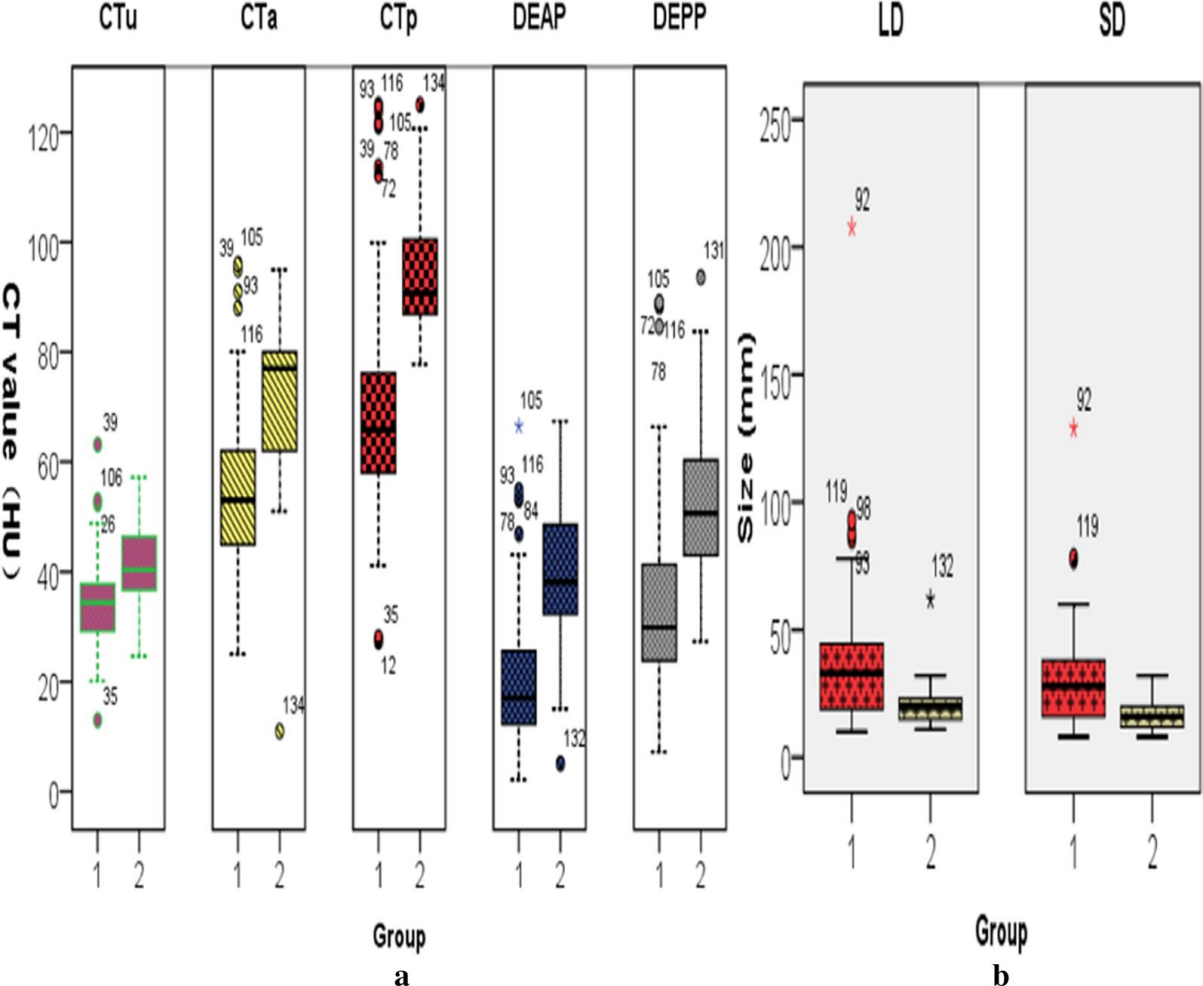
Clustered boxplot graph shows that the CT median attenuation value was universally higher for the GEPs than that of the GST, no matter what CTu, CTa, CTp, DEAP or DEPP, while the diameter was universally smaller

Table 3 showed the sensitivity and specificity values of each significant CT feature for differentiating GEPs from GST. According to the ROC analysis (Fig. 4), the largest area under the curve (AUC) was the CTp (0.900), followed by the DEPP (0.832), the CTa (0.821), the DEAP (0.806), the SD (0.757), the CTu (0.727) and the LD (0.726), which were significantly continuous variables differentiated GEPs from GST. Using clustered boxplot to study the significant continuous variables—CT attenuation value and diameter for differentiating GEPs from GST, we found the CT median attenuation value was universally higher for the GEPs than that of the GST, no matter what CTu, CTa, CTp, DEAP or DEPP, while the diameter was universally smaller, no matter LD or SD (Fig. 5).

**Table 3:** Sensitivity and specificity of significant CT features for differentiating GEPs from GST

CT features	Sensitivity	Specificity
Qualitative features		
Location at the body and antrum	94.1% (16/17)	42% (50/119)
Round or oval	88.2% (15/17)	39.5% (47/119)
Peritumoral infiltration or fat-line of peritumor	35.3% (6/17)	97.5% (116/119)
Intralesional necrosis absence	94.1% (16/17)	34.5% (41/119)
Intralesional calcification absence	100% (17/17)	19.3% (23/119)
Quantitative features		
LD (cut off: ≤ 25 mm)	82.4% (14/17)	79.0% (94/119)
SD (cut off: ≤ 22 mm)	94.1% (16/17)	59.7% (71/119)
CTu (cut off: ≥ 39.4 HU)	64.7% (11/17)	80.7% (96/119)
CTu (cut off: ≥ 39.4 HU)	70.6% (12/17)	88.2% (105/119)
CTp (cut off: ≥ 77.5 HU)	100 and (17/17)	76.5% (91/119)
DEAP (cut off: ≥ 32.2 HU)	76.5% (13/17)	84.0% (100/119)
DEPP (cut off: ≥ 42.5 HU)	82.4% (14/17)	79.0% (94/119)

*GEPs* gastric ectopic pancreas, *GST* astrointestinal stromal tumor, *LD* long diameter, *SD* short diameter; *CTu/CTa/CTp* the CT attenuation value of unenhancement phase/arterial phase/portal venous phase, *DEAP* the CT attenuation value of arterial phase-unenhanced phase, *DEPP* the CT attenuation value of portal venous phase-unenhanced phase

## Discussion

Gastric ectopic pancreas (GEPs) is a pancreatic tissue which was found outside its normal localization and without any anatomical or vascular connection with pancreas [3, 11]. Although the majority of patients with GEPs were asymptomatic, a few patients may have clinical manifestations due to stimulation of hormones and enzymes secreted by the ectopic pancreas [11]. As the most common subepithelial lesion, GST accounts for 90% of gastric submucosal tumor [8, 9], And it is difficult to differentiate GEPs from GST, so we compare the CT features of GEPs and GST to help us make the correct diagnosis.

Our study indicated that 0 (0%) of 17 cases of GEPs were located in gastric fundus and the majority of GEPs (64.7%) were located in gastric body, moreover, only 5 cases (29.4%) were located in gastric antrum. In contrast, 41 (34.4%) of 119 GST were located in gastric fundus and 56 (47.1%) cases were located in gastric body. However, the location of GEPs is inconsistent with the well-known fact that ectopic pancreases in upper gastrointestinal system are usually located in the gastric antrum [6–8, 12–14], and this may be resulted from mall sample size of GEPs.

Moreover, 6 (35.3%) of 17 cases of GEPs in our study had the presence of peritumoral infiltration or fat-line of peritumor, while only 3 (2.5%) of 119 cases of GST performed like that. Our result is similar to previous researches of Cho et al. [14] and Kim et al. [15] and consistent with typical endoscopic ultrasonography (EUS) characteristics, that is indistinct borders, lobulated margins, presence of anechoic duct-like structures, a mural growth pattern, and localization within two or more layers [16, 17]. The feature of peritumoral infiltration is very closely correlated with the histological feature of a lobular structure of the acinous tissue at the margin [16]. Since most GEPs were exophytic growth pattern (64.7% [11/17]) and GEPs was not a true neoplasms but a hamartoma that flat pancreatic acinar formation and duct development histologically [6–8], so it is of high possibility for GEPs to have fat space between the tumor and serosal layer. As for GST, the main endoscopic finding of it is a nonspecific smooth bulge covered with normal mucosa, which is common to all subepithelial lesions [9], so the possibility for GST to have fat space between the tumor and serosal layer is extremely low. Furthermore, Mietinenn [18] reported the metastatic risk of GST increases according to the tumor size irrespectively of the mitotic count and the probability of malignancy was significantly increased when the tumor was larger than 5 cm in diameter. In our series, 86 cases of GST (72.3% [86/119]) were less than 5 cm in diameter and it is of low probability for GST in our study to invaded the serosal layer, which lead to the seldom presence of peritumoral infiltration.

In our series, 0 (0%) of the 17 cases of GEPs had the presence of calcification, surface ulceration and lymph node, just 1 (5.9%) case of GEPs had necrosis, our results were supported by the fact that GEPs was not a true neoplasm but a hamartoma of flat pancreatic acinar formation and duct development [6–8]. Our study also showed that the LD and SD of GEPs were shorter than GST, but the previous study [14] regarding the CT features of GEPs didn’t regard it as a characteristic CT finding.

Our study demonstrated that the CT attenuation values of CTu, CTa and CTp of GEPs were significantly higher than that of GST. Besides, the degree of enhancement was much heavier for the GEPs than that of GST, both in the DEAP and DEPP. Furthermore, ROC analysis showed that the test efficiency of CTp in differentiating them was perfect (AUC = 0.9). The majority of GEPs appeared as homogeneously extramucosal masses with similar or higher attenuation to pancreas and this result may be attributable to the histologic similarity of GEPs to normal pancreatic tissue, especially acini. Microscopically, GEPs consist of pancreatic acini and ducts and rarely contain islet cells. If the GEPs was mainly composed of pancreatic acini, the lesions would show greater enhancement and have a higher CT attenuation value in portal venous phase than the pancreas. On the contrary, if the GEPs consisted of predominantly ducts, the lesions would have lower CT attenuation values than pancreas and even the back muscles [14, 19, 20]. Our result showed that 16 of 17 GEPs mainly consisted of pancreatic acini and 1 of 17 GEPs contained many ducts and a few acini, and it is similar to the report of Yamagiwa et al. [20]. But for GST, as we know, GST had malignant potential and fast growth rate so as to tumor cell prone to degeneration, which undoubtedly decreased the degree of enhancement of GST [9, 21].

Our study has several limitations. Firstly, this was a retrospective study, the selection bias and the use of various CT scanners were inevitable. Secondly, only two types of gastric mesenchymal tumors were compared and other subepithelial lesions were ignored. Besides, in order to avoid the influence of size bias, we excluded larger GST because most of larger-sized gastric mesenchymal tumors can be diagnosed as GST eventually [22]. Thirdly, our results were based on clinical data obtained from an eastern country at a single institution, which could not reflect fully the image differences between the different races and regions of two groups.

In conclusion, gastric ectopic pancreas had characteristic CT findings that differ from gastric stromal tumor. When characteristic CT imaging findings are used in combination, ectopic pancreas can be differentiated from with a high degree of diagnostic accuracy.

## Data Availability

The data underlying this paper are available upon request to the corresponding author due to ethical restrictions.

## Abbreviations

**GEPs**: Gastric ectopic pancreas
**GST**: Gastric stromal tumor
**CT**: Computed tomography
**CTu**: CT attenuation value of unenhancement phase
**CTa**: CT attenuation value of arterial phase
**CTp**: CT attenuation value of portal venous phase
**DEAP**: The CT attenuation value of arterial phase minus that of unenhanced phase
**DEPP**: The CT attenuation value of portal venous phase minus that of unenhanced phase
**LD**: Long diameter
**SD**: Short diameter
**ROC**: Receiver operating characteristic curve
**ROI**: Region of interest
**AUC**: Area under curve
